# Analysis of Selected Minerals in Homemade Grape Vinegars Obtained by Spontaneous Fermentation

**DOI:** 10.1007/s12011-021-02671-9

**Published:** 2021-03-25

**Authors:** Justyna Antoniewicz, Karolina Jakubczyk, Patrycja Kupnicka, Mateusz Bosiacki, Dariusz Chlubek, Katarzyna Janda

**Affiliations:** 1 Department of Human Nutrition and Metabolomics, Pomeranian Medical University in Szczecin, 24 Broniewskiego Street, 71-460 Szczecin, Poland; 2 Department of Biochemistry and Medical Chemistry, Pomeranian Medical University in Szczecin, 72 Powstańców Wlkp. Street, 70-111 Szczecin, Poland; 3 Department of Functional Diagnostics and Physical Medicine, Pomeranian Medical University in Szczecin, 54 Żołnierska Street, 71-210 Szczecin, Poland

**Keywords:** Mineral content, Elements, **Chaptalisation process**, **Sugar addition**

## Abstract

Fruit vinegars are widely used as a spice and food preservative. They are considered as functional food, containing many bioactive compounds with pro-health benefits. Grape vinegars can be also a source of mineral compounds. Their quantity and diversity can be determined by environmental factors and growing conditions, such as temperature, mineral composition of the soil, heavy metal contamination, sunlight availability as well as grape variety and fruit ripeness stage. The aim of the study was to determine the content of minerals in homemade grape vinegars, obtained by spontaneous fermentation. Five different grape (*Vitis vinifera* L.) varieties were used in the study (Cabernet Cortis, Johanniter, Solaris, Souvignier gris and Prior). Moreover, the effect of sugar addition in the fermentation process on the mineral content was examined. The mineral content was determined using the ICP-OES method. Among the analysed samples, potassium was the most abundant element (936.07–1472.3 mg/L of vinegar). Comparative analysis showed that the content of Ca, Fe and Cr was significantly higher in vinegars prepared from red varieties than in white-coloured ones. In turn, vinegars prepared from white grape varieties contained statistically significantly higher content of potassium. Vinegar colour did not have a significant influence on the content of the remaining elements included in the analysis. Furthermore, statistical analysis did not reveal any significant differences in the content of the analysed minerals in any of the grape varieties used between the samples with and without sugar addition.

## Introduction

Grape vinegars are fermented grape derivatives with typical characteristics regarding its aroma and flavour [1]. They are produced in a two-stage fermentation process [2] and practically any source with a high carbohydrate content can serve as a starting material for vinegar-making [3]. Vinegars are most commonly made from molasses, dates, apples and wine [4]. In the first stage of the process, yeasts convert fermentable sugars into ethanol [5]. Then, under aerobic conditions, acetic acid bacteria (AAB) transform ethanol into acetic acid [6]. The process also involves the formation of small amounts of other organic acids, including tartaric or citric acid, as well as esters, aldehydes and ketones [7]. Vinegars have been used for thousand years as a condiment and food preservative [8]. They have been also used in traditional folk medicine for lowering blood pressure and blood sugar levels, stimulating the digestive system and appetite [9], and also as a beauty product [10]. Grape vinegars are also considered as a functional food, i.e. providing biologically active ingredients with health benefits related to the prevention of chronic diseases [11]. Numerous researches have established that grapes and its products, such as grape vinegars, have anti-glycemic [12, 13], cardioprotective [14], neuroprotective and anti-inflammatory [15] properties.

In addition to many health-related aspects resulting from the consumption of grapes and products prepared from them, their importance as a source of mineral compounds should also be emphasised [16]. Their content in plant material, including grapes and their derivative products, is strongly dependent on environmental factors and growing conditions, i.e. temperature, mineral composition of the soil, contamination with heavy metals [17], amount of precipitation, exposure to sunlight as well as the use of plant protection products and fertilisers [18]. Other important factors include the grape variety [19] and ripeness of the fruit [20]. The elemental content in vinegar will also be affected by substances arising during fermentation and maturation processes, and the equipment used in vinegar production and storage [21]; therefore, the elements found in grape derivatives such as wine or vinegar can have an exogenous and an endogenous origin [22]. It has to be remembered that mineral compounds found in vinegar perform various functions in humans and play a key role in a variety of processes necessary for life throughout biochemical reactions [23, 24]. While some minerals are essential for human nutrition (e.g. Fe, Cu, Se and Zn) [25], excessive amounts of certain elements, notably heavy metals found in grapes, including Pb, may be potentially harmful to human health, e.g. by damaging DNA [26]. Additionally, some minerals in low concentrations are essential for the human body to function normally, but in greater amounts may pose a health hazard [27].

In wine-making, it may be allowed to add sugar during fermentation, in a process known as chaptalisation. The purpose of the procedure is to increase the ethanol content or to obtain a sweeter flavour in the final product [28]. Some studies have suggested that chaptalisation impacts the levels of macro- and micronutrients in wine [29].

However, there is a lack of studies concerning the mineral content of homemade grape vinegars to provide valuable information for correctly designing grape vinegar production and chaptalisation with the corresponding grape at home conditions.

Data on the mineral content in wines have been widely studied and reported [30–32]. Various studies indicate that trace element composition can be used to fingerprint wines and reflect the provenance or region of origin [33, 34]. There is also an increasing emphasis on the significant correlation between mineral content and the wine production method [35]. However, in the case of grape vinegars and other non-wine grape derivatives, only a few studies on mineral content are found [21, 36, 37].

The aim of this study was to determine the content of macro- and micronutrients in homemade grape vinegars from different grape varieties cultivated in the northwestern part of Poland. Additionally, the authors examined the effects of chaptalisation and colour of the fruit on the elemental content of minerals included in the analysis.

## Materials and Methods

### Grape Vinegars

The vinegars used in the study were made from the fruit of wine grape varieties (*Vitis vinifera* L.) obtained from a vineyard in the Western Pomerania (Zachodniopomorskie) region of Poland (53° 15′ 35″ N 14° 43′ 24″ E) in September 2018. The study used white grape varieties, Solaris, Johanniter and Souvignier gris, as well as red grape varieties, Prior and Cabernet Cortis. Grapes were collected from individual plants of each variety during the full ripening stage of the grape. For each grape variety, vinegars were prepared according to two different procedures. In variant 1, only crushed fruit and distilled water were used at a 1:1 ratio. In variant 2, additionally, the chaptalisation process was used: a solution of distilled water and table sugar (70 g sugar per 1 L of water) was added to the fruit (also 1:1 ratio). Whole grape fruits with skins and seeds were used to prepare the vinegar (the stalks were removed). Vinegars were produced in glass jars by spontaneous fermentation at room temperature (24°C) over a period of 2 months, carried out by the natural flora inhabiting the fruit. Both variants of the fermentation process were performed in triplicate.

### Determination of the Mineral Content

#### Sample Preparation

The samples were mineralised using the CEM MARS 5 microwave digestion system. The sample volume was 0.8 mL. The samples were transferred to clean polypropylene tubes. A total of 0.6 mL of 65% HNO_3_ (Suprapur, Merck, Darmstadt, Germany) was added to each vial, and each sample was allowed 30-min pre-reaction time in the clean hood. At the end of the pre-reaction time, 0.6 mL of non-stabilised 30% H_2_O_2_ solution (Suprapur, Merck, Darmstadt, Germany) was added to each vial. After all reagents were added, the samples were placed in special Teflon vessels and heated in the microwave digestion system for 35 min at 180 °C (15-min ramp to 180 °C and maintained at 180 °C for 20 min). At the end of digestion, all samples were removed from the digestion oven and allowed to cool to room temperature. In the clean hood, samples were transferred to acid-washed 15-mL polypropylene sample tubes. A further tenfold dilution was performed prior to inductively coupled plasma optical emission spectrometry (ICP-OES) measurement. The volume of 1 mL was taken from each digest. The samples were spiked with an internal standard to provide a final concentration of 0.5 mg/L yttrium, 1 mL of 1% Triton (Triton X-100, Sigma-Aldrich, USA) and diluted to a final volume of 10 mL with 0.075% nitric acid (Suprapur, Merck, Darmstadt, Germany). Blank samples were prepared by adding concentrated nitric acid (500 μL) to tubes without the sample and subsequently diluted in the same manner as described above. Multi-element calibration standards (ICP multi-element standard solution IV, Merck) were prepared with different concentrations of inorganic elements in the same manner as in blanks and samples. Deionised water (Direct Q UV, Millipore, approximately 18.0 MΩ) was used in the preparation of all solutions.

#### Sample Determination

All samples were transferred into tubes and stored at −20 °C until processed. Samples were analysed by ICP-OES (ICAP 7400 Duo, Thermo Scientific). This method is often used to measure the concentration of elements in plant tissues [38, 39]. ICP-OES with a concentric nebuliser and cyclonic spray chamber was used to determine the content of micro and macroelements.

The analysis was performed in both radial and axial modes. Validation was performed by evaluating the following: NIST SRM 8414 reference material (Bovine Muscle Powder, National Institute of Standards and Technology, USA), limit of detection (LOD), relative sample deviation (%RSD) range and the recovery of internal standard (yttrium) (Table 1). To eliminate possible interference, the emission lines were selected empirically in pilot measurement. This model of validation is often used in ICP-OES studies, also those regarding plant samples [39]. The recovery of Y was within 89–105%. The *R*^2^ values for all standard curves were in the range between 0.998 and 1.000.

**Table 1:** Analysis of reference material Bovine Muscle NIST-SRM 8414, limits of detection (LOD) and relative sample deviation (%RSD) range

Element	Certified (mg/L)	Measured (mg/L) (*n*=3)	LOD (mg/L)	%RSD range
Ca	145 ± 20	141	0.00676	0.4–2.5
Mn	0.37 ± 0.09	0.43	0.00026	1.0–6.2
K	15170 ± 370	15290	0.08426	0.3–1.4
Zn	142 ± 14	138	0.00065	1.5–5.7
Cu	2.84 ± 0.45	3.06	0.00186	2.4–8.2
Fe	71.2 ± 9.2	76.1	0.00022	1.8–6.7
Na	2100 ± 80	2146	0.08137	0.8–4.1
Pb	0.38 ± 0.24	0.48	0.00178	5.3–11.2
Cr	0.071 ± 0.038	0.080	0.00044	3.9–9.2
P	8360 ± 450	8874	0.00532	0.8–3.2
Mg	960 ± 95	923	0.00159	0.5–1.9

### Statistical Analysis

Each type of vinegar has been prepared in triplicate. Additionally, during the analysis of the content of elements, each sample was tested three times. The statistical analysis was performed using StatSoft Statistica 13.0 and Microsoft Excel 2017. The results are expressed as mean values and standard deviation (SD). Distributions of values for each parameter were analysed using the Shapiro-Wilk test. To assess the differences between examined parameters, one-way analysis of variance (ANOVA) with Tukey’s post hoc test was used. Differences were considered significant at *p*≤ 0.05.

## Results and Discussion

### Mineral Concentrations in Vinegars

Twelve mineral elements were identified and quantified in the analysed samples of grape vinegars (Table 2). The elemental analysis revealed the presence of many macro- and microelements as well as trace elements. The general scheme of ascending concentrations of elements in the studied vinegar samples was as follows: K > P > Ca > Mg > Na > Zn > Mn > Fe > Sr> Pb> Cu> Cr.

**Table 2:** Elemental composition of vinegar samples

Vinegar sample	Element (mg/L)											
	Ca	Mn	K	Zn	Cu	Fe	Na	Pb	Cr	Sr	P	Mg
J^1^	74.73±18.09	0.56±0.01^3,4,10^	1141.80±73.36	2.14±1.49	0.14±0.01^7^	0.46±0.02	13.91±0.69	LOD	0.037±0.01	0.21±0.04	113.87±5.86^7,8^	62.64±4.15
J^(ch)2^	65.82±12.02^3,9,10^	0.59±0.02^3,10^	1060.96±92.22	0.60±0.13	0.18±0.04	0.45±0.07	13.34±2.03	0.09±0.01	0.031±0.01	0.21±0.06	113.88±0.96^7,8^	62.79±7.40
C^3^	125.91±14.20^2^	1.16±0.23^7,8^	1247.92±98.93	1.29±0.47	0.23±0.01	0.64±0.18	15.95±2.47	0.08±0.06	0.081±0.01	0.46±0.07	173.58±1.30	81.36±3.11
C^(ch)4^	105.42±6.38	1.13±0.09^7,8^	1127.20±113.09	1.33±0.55	0.23±0.05	0.57±0.03	14.20±1.16	LOD	0.056±0.01	0.35±0.01	159.07±20.78	68.52±6.23
S^5^	112.15±30.61	1.06±0.17^7,8^	1224.72±123.11	1.50±0.05	0.26±0.08	0.49±0.00	17.16±6.33	LOD	0.068±0.03	0.50±0.10	134.87±40.13	96.09±21.23
S^(ch)6^	118.9±41.60	1.05±0.09^7,8^	1302.61±25.65	1.39±0.41	0.26±0.04	0.55±0.06	17.20±2.09	0.11±0.02	0.014±0.01	0.51±0.02	138.69±20.35	100.5±19.17
P^7^	108.0±1.49	0.45±0.01^3,4,5,6,10^	1472.30±26.30	1.37±0.15	0.27±0.01^1^	0.63±0.02	11.62±0.62	0.58±0.12	0.036±0.01	0.56±0.02	212.71±1.47^1,2^	66.94±0.69
P^(ch)8^	101.7±13.74	0.44±0.04^3,4,5,6,10^	1461.66±237.81	1,45±0.69	0.33±0.02	0.48±0.12	10.95±1.52	LOD	0.023±0.01	0.50±0.07	214.08±41.01^1,2^	66,21±10.22
G^9^	148.5±9.59^1,2^	0.95±0.30	936.07±84.77	2.03±0.59	0.23±0.11	0.65±0.08	14.39±0.71	0.46±0.04	0.047±0.01	0.55±0.06	152.24±2.40	81.45±0.70
G^(ch)10^	127.0±3.67^2^	1.19±0.02^2,7,8^	945.66±160.58	1.00±0.01	0.14±0.04	0.81±0.19	12.61±0.21	LOD	0.076±0.03	0.48±0.00	134.26±11.95	65.75±3.02

Data represent the mean values ± standard deviations of the three biological × three technical replicates. Different numbers in superscript represent statistically significant differences in elements (*p*≤0.05). *LOD* limit of detection (LOD for Pb and Cr = 0.01 mg/L)

*J* Johanniter, *C* Cabernet Cortis, *S* Solaris, *P* Prior, *G* Souvignier gris

*(ch)* chaptalisation process

Potassium, phosphorus, calcium, magnesium and sodium were the most abundant elements. These minerals are essential for normal growth of the grapevine [40]; hence, their high levels in vinegars are to be expected. Potassium (K), as the predominant cation in grape derivatives [41], was the element found in the highest concentration (936.07–1472.3 mg/L of vinegar). However, statistically significant differences did not exist. The highest concentrations of potassium in all the studied vinegars were also observed by Akpinar-Bayizit et al. [42], but the levels observed by those researchers were much lower than ours (710.19 ± 310.659 mg/L). Our findings do not confirm the claim that K levels in grapes grown in a hot climate considerably exceed those in grapes from colder climate zones [43]. The levels observed in our study are similar to the potassium content in Andalusian wine vinegars (K content 372–1814 mg/L) [44]. The phosphorus content in the analysed samples ranged from 113.87 to 214.08 mg/L. Significant statistical differences in the content of this element were observed between vinegar Johanniter vs. Prior, *p*=0.01538; Johanniter vs. Prior with sugar, *p*=0.00336; Johanniter with sugar vs. Prior, *p*=0.02305; and Johanniter with sugar vs. Prior with sugar, *p*=0.02807. The content of this element depends on the mineral composition of the soil in the place of cultivation and the plant’s assimilation ability [45]. When analysing the country of origin of the vines, a disproportion is observed. P content in Portuguese wines according to Cabrita et al. [22] is 281.57 mg/L for red wines and 250.39 for white wines. In turn, Andalusian wine vinegars contained from 51.32 to 219 mg/L of phosphorus [44]. Calcium and magnesium are, cooperatively with potassium, the elements most absorbed by grapevine [46]. The highest level of calcium was observed in the vinegar made from Souvignier gris grapes with no added sugar (148.5 mg/L), and the lowest in the Johanniter vinegar with added sugar (65.82 mg/L). The highest level of magnesium was observed in the vinegar made from Solaris grapes with added sugar (100.05 mg/L) and the lowest in the Johanniter vinegar without added sugar (62.64 mg/L). The levels of those minerals depended on grape variety, which is also confirmed in the literature [37]. Despite the differences in the levels of those minerals in the tested samples, statistically significant differences were observed only for the Ca content in some vinegar samples (Johanniter with sugar vs. Cabernet Cortis, *p*=0.02518; Johanniter with sugar vs. Souvignier gris with sugar, *p*=0.01904; Johanniter with sugar vs. Souvignier gris with sugar, *p*=0.01216; and Johanniter vs. Souvignier gris, *p*=0.01508). The Ca and Mg levels determined in our study were comparable to those observed in wines by Kment et al. [47]. The authors conducted an elemental analysis of wines from the Czech Republic, which belongs to the same wine-growing zone as Poland. The Ca content in the study fell in the range of 47.7–210 mg/L, and that of Mg is 48.9–108 mg/L.

The vinegars were also tested for concentrations of micronutrients, such as Fe, Mn, Zn and trace elements Cu and Sr. Other heavy metals, such as Pb and Cr, were also measured in this study; however, the concentrations of those elements in the digested samples failed to reach the detection limit (LOD) by ICP-OES (Pb LOD 0.01 mg/L, Cr LOD 0.01 mg/L). The lowest concentration of iron (Fe) in the tested samples was found in the Johanniter vinegar without added sugar (0.45 mg/L), and the highest in the Souvignier gris vinegar without added sugar (0.81 mg/L); however, significant statistical differences do not exist. The Fe content in wines and other grape products is strongly correlated with the soil levels of that mineral at the growing site [48]. It can also be affected by the methods used to obtain grape products, including fermentation processes and vinification equipment [49]. Please note that in the above study, all grape varieties originated from a single geographical region; hence, it may be concluded that the soil levels of that mineral would be similar.

The highest level of Mn was observed in the vinegar made from Souvignier gris grapes with added sugar (1.19 mg/L), and the lowest in the Prior vinegar with added sugar (0.44 mg/L). The Zn content ranged from 0.6 mg/L (Johanniter with added sugar) to 2.14 mg/L (Johanniter without added sugar). In the analysis of copper (Cu) levels, the lowest level was noted in the vinegar made from Johanniter grapes without added sugar (0.14 mg/L), and the highest in the Prior vinegar with added sugar (0.33 mg/L). In turn, the content of strontium (Sr) ranged from 0.21 mg/L (Johanniter with added sugar) to 0.56 mg/L (Prior without added sugar). Statistical analysis showed no significant differences in the content of any of the above elements in the tested vinegar samples.

Elements such as Mn, Zn and Cu can be found in plant protection products, fertilisers, pesticides and fungicides. Their presence in the analysed samples may be related to the agricultural practices in use [20] and the content of those elements in phytosanitary products [28, 50]. Notably, Cu levels may be affected by products containing copper sulphate, which are sprayed on grapevines to prevent mildew [48]. In our study, all the tested samples originated from the same growing location and were subjected to the same agricultural practices. The differences in the content of Cu and Mn in the respective varieties were statistically insignificant (*p*≤0.05) (Table 2). In the case of Zn content, no statistically significant differences were observed between the analysed samples.

Lead (Pb) tends to be present in elevated concentrations in grapevines grown in the vicinity of roads or industrial areas [51]. Its content in some samples may also be associated with the presence of lead in glass containers. Carvalho et al. observed that for some glass containers extended storage periods were positively correlated with higher Pb levels in the analysed wine samples [52]. In the present study, Pb content was distinctly varied, but in the majority of samples, the level of that mineral could not be determined because it was below the detection level (0.01 mg/L). In the majority of the successfully detected samples, the Pb content did not exceed 0.1 mg/L. Clearly, higher Pb levels were observed in the vinegars made from Souvignier gris grapes (0.46 mg/L) and Prior grapes (0.58 mg/L). Our findings with regard to the concentrations of lead were significantly higher than those observed by Ndung’u et al. [53], where Pb content in wine vinegars fell in the range of 0.036–0.05 mg/L. Such a high lead content may point to serious contamination at the growing site with this element. The phenomenon warrants further investigation. Nevertheless, these figures are still below the limit for Pb content in vinegar established in the Codex Alimentarius, which amounts to 1 mg/L [54]. It is worth highlighting that vinegars made from Prior grapes also contained the highest levels of copper and strontium (respectively 0.27± 0.01 mg/L and 0.56± 0.02 mg/L for the variant without added sugar and 0.33± 0.02 mg/L and 0.50± 0.07 mg/L for the variant subjected to chaptalisation). In turn, the highest Cr content in the tested samples was found in the vinegar made from Cabernet Cortis grapes (without added sugar) (0.081 ± 0.01 mg/L).

Determination of the content of micronutrients and toxic elements is important to set the quality and health safety of the product [47] [55]. The levels of individual macro- and micronutrients in grape vinegars may depend on the natural presence of those minerals in the grapevine, environmental contaminants and release from the apparatus used in fermentation processes [56].

When comparing the mineral levels determined in our study with the findings obtained by other researchers, one must bear in mind that Poland, along with Germany, Austria and Czech Republic, belongs to the coldest zone of grapevine cultivation in Europe. The organoleptic characteristics of grapevine products grown in cold climates differ from those cultivated in traditional wine-producing countries [57].

### The Impact of Grape Colour on the Mineral Content in Vinegars

**Fig. 1:**
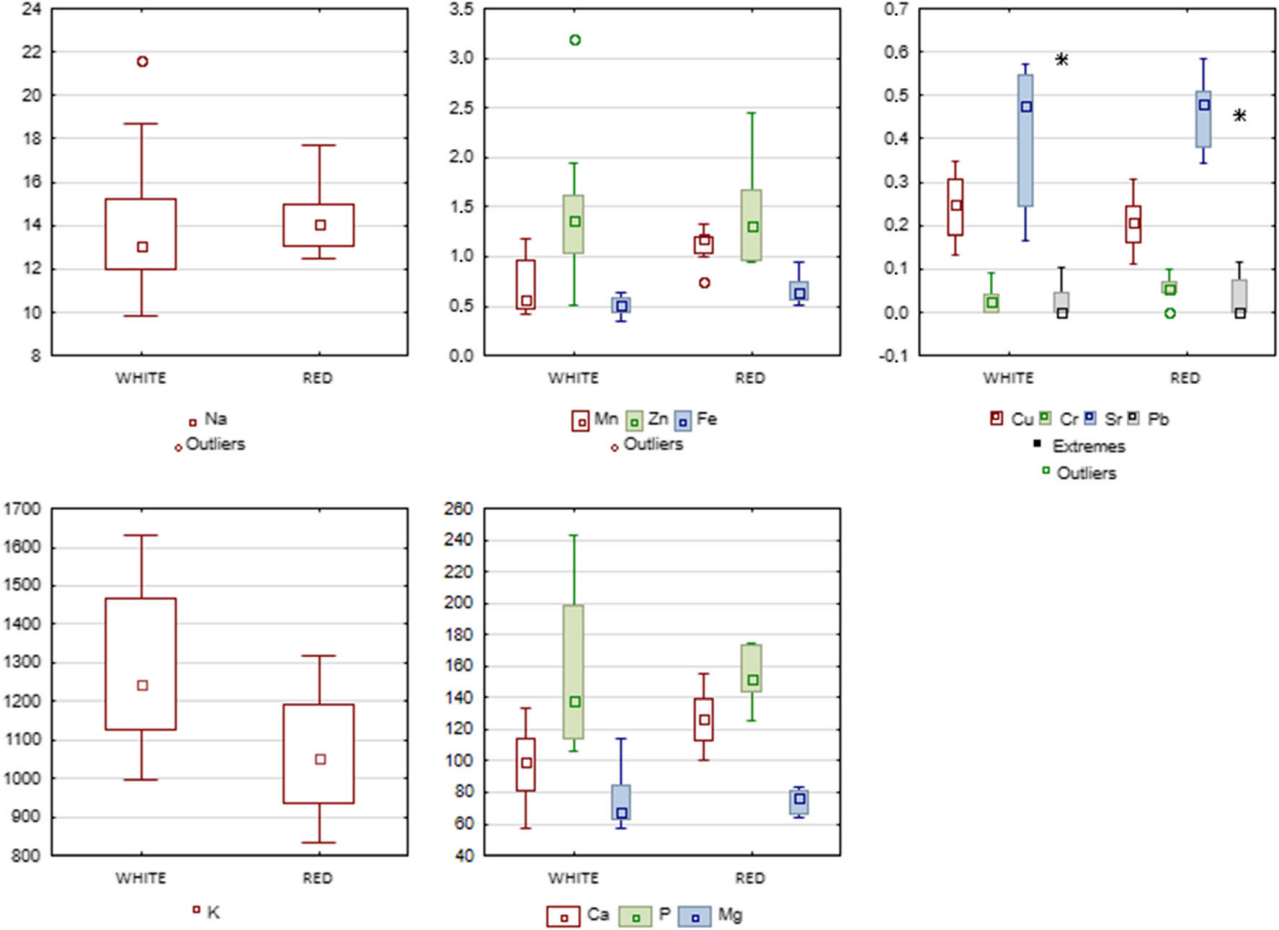
Box plots of concentration of elements (expressed by means, standard deviations, outliers and extremes) in the vinegars made from different varieties depending on colour. Results are expressed as mg/L

Our study included a comparative analysis of elemental content between all white vinegars (Solaris, Johanniter and Souvignier gris) and red-coloured vinegars (Prior and Cabernet Cortis) (Table 3). It was observed that the content of Ca, Mn, Fe and Cr was significantly higher in red-coloured vinegars (respectively *p*=0.0121, *p*=0.0029, *p*=0.0151, *p*=0.0440). On the other hand, the potassium content was significantly higher in white-coloured vinegars (*p*=0.04910). Box plots with concentrations of analysed minerals in vinegars depending on the colour of the fruit are presented on Fig. 1. Vinegar colour did not have a significant influence on the content of the remaining elements included in the analysis. A study comparing the mineral contents in juices made from white and red grape varieties conducted by Dani et al. revealed significantly higher Mg levels in juices made from red-coloured varieties compared to white, as well as a statistically significantly higher content of Cu in juices made from white grape varieties [58]. It needs to be noted, however, that the differences in mineral content compared to this study may potentially be due to the fact that the researchers had used juices obtained from different varieties of *Vitis labrusca*. In turn, *V. vinifera* was studied by Olalla et al., who found a significantly higher Zn content in grape juice samples made from red-coloured fruits, which could not be confirmed in our study. Additionally, the levels of that element in juice samples were much lower than those determined in our study (0.55 mg/L for red grape juice and 0.41 mg/L for white grape juice) [59].

**Table 3:** Mineral composition of grape vinegars depending on grape colour

Grape vinegar	Mineral content (mg/L)											
	Ca	Mn	K	Zn	Cu	Fe	Na	Pb	Cr	Sr	P	Mg
Red grape vinegars	127 ± 17.75	1.11±0.178	1064±166	1.413±0.539	0.205±0.0618	0.669±0.142	14.29±1.658	0.203±0.224	0.063±0.0203	0.459±0.0834	155±17.65	74.27±8.219
White grape vinegars	96.9 ± 23.86*	0.693±0.279*	1277±198	1.409±0.695	0.24±0.0742	0.51±0.090*	14.03±3.345	0.261±0.280	0.0367±0.0280*	0.416±0.1590	155±48.20*	75.86±19.22

Data are reported as means ± SD; *p<0.05 for differences between red and white grape vinegars

Visible differences in the elemental content between white and red grape varieties are often observed in the case of wines. Studies report significantly higher levels of macro- and micronutrients in red wines compared to white wines [32]. These differences can be attributed to the dissimilar production processes, whereby in the red wine fermentation process grape skins remain in contact with must for longer than in the production of white wines [43]. This correlation has been borne out in research, including the study by Cox et al. Their analysis of white and red Californian wines revealed significantly higher levels of Na (respectively, 131 mg/L for red wines and 50.2 mg/L for white wines) and Fe (8.93 mg/L for red wines and 5.31 mg/L for white wines) [60]. Likewise, Martin et al. observed that red wines had a much higher content of Mg, P and K than white wines [61]. Conversely, a comparative analysis conducted by Semla et al. on a range of Slovakian wines (white, rosé and red) found that the differences in the levels of analysed minerals were not statistically significant [62].

### The Impact of Chaptalisation on the Mineral Content in Vinegars

Chaptalisation is the process of adding sugar during wine production. The purpose of the procedure is to increase the ethanol content in the final product by increasing the amount of substrate for the yeast to ferment. Chaptalisation is employed when must does not contain sufficient amounts of alcohol, which may be the case when fermenting fruit which has not fully ripened [43, 63], e.g. in the colder climates of northern Europe [64]. Depending on the region, the process is strictly regulated or prohibited altogether [65]. Poland, along with countries like the Czech Republic, Belgium and Germany, belongs to the coldest wine-growing region [66], recognised as zone A. pursuant to Regulation (EU) No. 1308/2013 of the European Parliament and of the Council, permits the use of chaptalisation in wine-making in this zone [67].

**Fig. 2:**
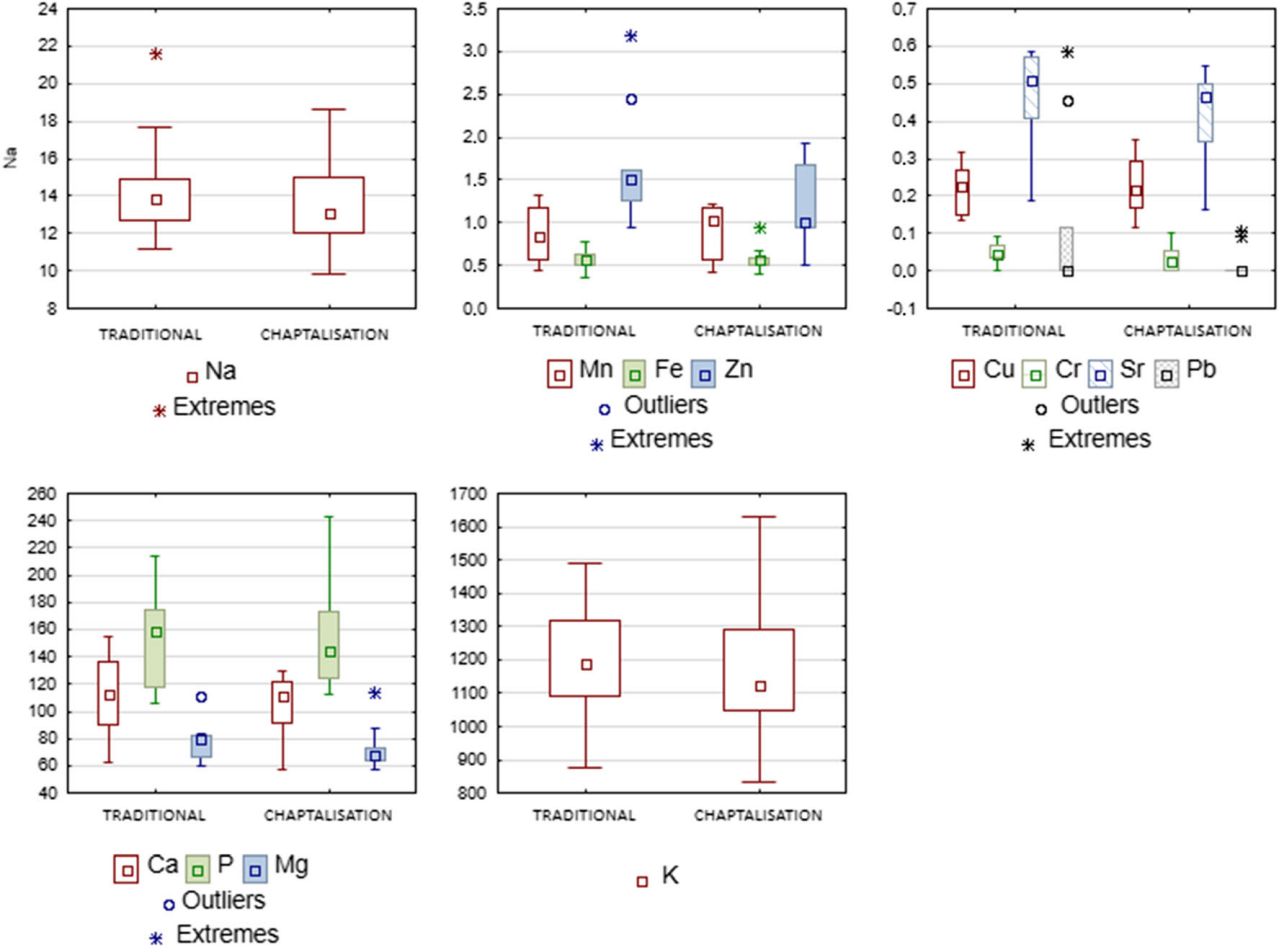
Box plots of concentration of elements (expressed by means, standard deviations, outliers and extremes) in the vinegars made with 7% chaptalisation process and with traditional method. Results are expressed as mg/L

Our study included a comparative analysis of elemental content between samples of vinegar obtained by fermentation without additives and those obtained with the use of chaptalisation. The results are presented in Table 4. Figure 2 presents box plots with concentrations of analysed minerals in vinegars depending on the chaptalisation process. Factors impacting on the mineral content in the samples subjected to chaptalisation include the formation and precipitation of insoluble elemental complexes related to an increased alcohol concentration [40]. However, the differences in the levels of the analysed elements observed in our study were too small to conclude whether they were due to the addition of sugar. Furthermore, statistical analysis did not reveal any significant differences in the content of the analysed minerals in any of the grape varieties used. Vinegars were chaptalised using refined sugar—sucrose, the purest form of sugar which should not contain any minerals potentially affecting the contents of the macro- and micronutrients under examination. In contradiction, Shimizu et al. observed that chaptalisation had a significant impact on the content of macro- and micronutrients in Koshu wine. A particularly striking increase was reported with respect to the level of sodium, which in the case of 10% chaptalisation was nearly doubled compared to the control sample. On the other hand, the addition of sugar caused a decline in the content of other minerals, e.g. molybdenum [29], which may have been due to the formation of insoluble complexes including tartrates, proteins and polysaccharides [40].

**Table 4:** Mineral composition of grape vinegars depending on chaptalisation process

Grape vinegar	Mineral content (mg/L)											
	Ca	Mn	K	Zn	Cu	Fe	Na	Pb	Cr	Sr	P	Mg
Produced traditionally	114±28.6	0.838±0.325	1205±194	1.667±0.671	0.224±0.067	0.574±0.119	14.61±3.04	0.298±0.264	0.0507±0.0261	0.456±0.140	157±38.21	77.69±14.51
With chaptalisation	104±23.2	0.882±0.326	1180±235	1.154±0.469	0.228±0.076	0.573±0.158	13.66±2.47	0.0995±0.008	0.0452±0.0321	0.410±0.128	152±39.96	72.76±16.78

Data are reported as means ± SD

## Conclusion

Our findings clearly show that the variety of grapes chosen as the material in vinegar-making had a significant impact on the mineral content in the final product. Furthermore, the colour of fruit determined the content of macro- and micronutrients in the final product, too. Our study revealed that vinegars made from red grape varieties contained statistically higher amounts of Ca, Mn, Fe and Cr, while K concentration was markedly higher in white-coloured vinegars. The use of chaptalisation, on the other hand, did not have a significant effect on the levels of minerals included in the analysis. While on a daily basis they are consumed in small amounts, vinegars obtained by spontaneous fermentation of grapes cultivated in Poland may serve as one of the sources of minerals in human diet.

## Data Availability

Not applicable
